# The Roles of Age and Depression in Affective Responsivity to Daily Positive Events

**DOI:** 10.21203/rs.3.rs-10000529/v1

**Published:** 2026-06-29

**Authors:** Zachary E. Taylor, Sumner J. Sydeman, Ann H. Huffman, Eric S. Cerino

**Affiliations:** 1 Department of Psychological Sciences, Northern Arizona University; 2 Center for Community Health and Engaged Research, Northern Arizona University

**Keywords:** Mood Brightening, Depression, Daily Positive Events, Daily Affect, Aging, Lifespan Development, Intraindividual Variability

## Abstract

Depression can present severe challenges in daily functioning for individuals across the adult lifespan. Growing older is associated with strengths in emotion regulation (ER) and physiological challenges that can influence depressive symptoms. Affective responsivity (changes in levels of positive affect (PA) or negative affect (NA) in response to an event) may be an indicator of ER disturbances and emotional instability in individuals with depression where previous research has found a “mood brightening” (MB) pattern characterized by increased affective responsivity to positive events. Past work, however, primarily examines MB in younger adult samples and does not evaluate age-related patterns. Using waves 2 and 3 of the Midlife in the United States Study and National Study of Daily Experiences, we examined cross-sectional baseline age differences and longitudinal changes in MB. The analytic sample comprised of 2,022 participants (M_age_=58.61 years, SD=12.12, Range=35–86; 10.63% screened positive for depression). Across 8 consecutive days in ~2005 and ~10 years later, participants reported on their positive affect, negative affect, and frequency of positive events. Multilevel models evaluated age patterns in MB with statistical adjustment for sociodemographic and structural covariates. MB was amplified among older adults such that older adults who screened positive for depression had more PA responsivity to positive events than younger adults. Further, the magnitude of MB decreased across the 10-year follow-up, especially for comparatively older adults in the sample. Results are discussed with implications for identifying potential early daily markers of depression risk and informing personalized, age-inclusive healthcare approaches.

Depression can present severe challenges in daily functioning for individuals across the adult lifespan. In 2020, 8.4% of US adults experienced a major depressive episode (i.e., a period of two or more weeks where individuals reported feeling depressed or that they lost interest in most things, and several other symptoms (American Psychiatric Association, 2013). The rates were highest for adults aged 18–25 (17.0%). Rates decreased with age, with adults aged 26–49 having a prevalence of 9.1%, and adults over 50 having a prevalence of 5.4%. Another study of a diverse sample of 175,956 adults over age 65 in the US found that 10.2% of the sample screened positive for depression (Hooker et al., 2019). Participants 85 and older reported the highest rate among the sample, with 13.63% screening positive. These findings highlight the importance of understanding unique age-related patterns of emotion dysregulation in depression. Increasing understanding of this topic can help inform tailored approaches for interventions at different developmental phases across the adult lifespan.

## Emotion Regulation and Dysregulation in Depression

Depression is characterized by emotion dysregulation. Emotion regulation has been described in a five-component process whereby an individual 1) encounters an event, 2) responds to the event behaviorally in ways that can modify the situation, 3) chooses to direct attention to specific aspects of the event, 4) constructs a cognitive meaning about the event based on previous steps in the process, and then 5) experiences an emotional response (e.g., affective responsivity; John & Gross, 2004). Depression is associated with a reduced use of more effective emotion regulation strategies such as cognitive reappraisal and increased use of suppression of emotion (Joorman & Stanton, 2016).

## Affective Responsivity and Mood Brightening

Affective responsivity may serve as a distinct marker of emotion dysregulation. Affective responsivity refers to changes in positive affect (PA) or negative affect (NA) in response to an event. Daily affective changes and reactions are often examined in response to day-to-day stressors and have been found to predict depression up to 10 years later (Charles et al., 2013; Stawski et al., 2022). Synthesis of these findings and developmental theory suggests that affective responsivity may be useful in clinical contexts as a potential early marker of increased depression risk that can be the target of early interventions that build emotion regulation skills. Much of the literature focuses on *negative* affective responses to daily *stressors* (e.g., Almeida et al., 2022; Charles et al., 2013; Stawski et al., 2022), with less focus on *positive* affective responses to daily stressors. Even fewer studies have examined either positive or negative responsivity to daily *positive events*. The studies that have examined responsivity to daily positive events have consistently found a phenomenon known as mood brightening (MB) in individuals with depression. While overall levels of PA are generally lower in individuals with depression compared to people without depression (Heininga & Kuppens, 2021), these studies have found that individuals with depression tend to show higher responsivity (larger increases in positive affect, larger decreases in negative affect) to daily positive events than people without depression (Bylsma et al., 2011; Khazanov et al., 2019; Panaite et al., 2019; Peeters et al., 2003). These findings run contrary to Emotion-Context Insensitivity (ECI) theory which predicts blunted affective responsivity to positive events (Rottenberg et al., 2005). A recent study replicated this MB effect in daily life using the National Study of Daily Experiences (NSDE; Mukherjee et al., 2023). However, this work did not formally examine the role that age (and longitudinal changes) may play in the MB effect. The purpose of the current study is to directly extend this work by examining age patterns in MB using the NSDE.

## Strength and Vulnerability Integration Model

The strength and vulnerability integration model (SAVI) describes age-related strengths in emotion regulation as well as unique vulnerabilities (Charles, 2010; Charles & Piazza, 2024). Compared to younger adults, older adults report higher emotional well-being (Charles et al., 2023), better emotion regulation (Carstensen et al., 2000), less stressor reactivity (Birditt et al., 2005; Neupert et al., 2007), shorter duration of negative affect (Charles et al., 2016) and fewer interpersonal tensions (Birditt et al., 2005). However, older adults with four or more chronic conditions report levels of stressor reactivity that are just as high as in younger adults (Piazza et al., 2007). Together these findings help explain why there is an overall decline in depression across the lifespan. However, they also identify unique vulnerabilities in older adults that are experiencing chronic depressive symptoms. SAVI outlines that chronically sustained stressors (e.g., depression), may overwhelm an older adult’s ability to redirect attention away from negative stimuli and compound with lower physiological flexibility to create a unique risk for negative outcomes.

## The Current Study

Using a lifespan developmental approach to examine age patterns in MB, this study sought to answer two research questions. First, are there age differences in the magnitude of MB? We hypothesized that there would be age differences in MB, but did not make a directional hypothesis because older age could facilitate better emotion regulation strategies in daily life (leading to more attenuated affective responsivity and reduced MB effects) or older age could correspond with heightened vulnerabilities to sustained stressors (e.g., depression) that amplify MB. Second, does the magnitude of MB change over ten years? Using a similar rationale and non-directional expectation, we hypothesized that the magnitude of MB would change over ten years. Growing older is linked with improvements in emotion regulation and declines in stressor reactivity (Birditt et al., 2005; Carstensen et al., 2000). However, aging is associated with higher risk for chronic conditions, which may counteract age-related improvements and strengths in emotion regulation. Investigating longitudinal change separately from cross-sectional age differences will allow the current study to more thoroughly examine longitudinal changes distinctly from baseline cross-sectional age differences. Additionally, we conducted exploratory analyses to evaluate whether different age groups exhibit different trajectories of longitudinal change across the ten-year follow-up.

## Method

### Transparency and Openness

Data are publicly available at the following website: https://www.icpsr.umich.edu/web/ICPSR/series/203. All analyses were completed using SAS 9.4 (SAS Institute, 2013). Research questions, hypotheses, and analysis plan were preregistered at https://osf.io/f4xeh. MIDUS and NSDE were approved by the Institutional Review Board of the institution responsible for data collection, and all respondents consented to their participation. The current study’s secondary data analysis was designated as non-human subjects research and was approved by the institutional review board at the authors’ university (IRB project number: 2069152–1).

### Participants and Procedure

The data used in this study comes from the Midlife in the United States Study (MIDUS) Survey project and the National Study of Daily Experiences (NSDE), which consists of a randomized subsample of the total MIDUS sample (Almeida et al., 2025). Participants were recruited across the US through random-digit dialing to achieve a national probability sample.

Respondents who agreed to participate completed the MIDUS survey which included information on demographics (including age) and 12-month prevalence of depression. This survey was repeated approximately every ten years with the first wave of data collection including an adult lifespan-representative sample of 7,108 participants. To date, three waves of data have been collected as a part of MIDUS. At the first wave in ~1995, a random 1,499 participants that completed the MIDUS survey were then invited to complete the NSDE, where they completed a daily diary over the phone every night for eight days covering experiences that occurred throughout the day. At the second wave in ~2005, an additional 1,048 participants were added to the NSDE. 974 participants from the first wave also completed the second wave of the NSDE, totaling up to 2,022 participants eligible for participation in the current study (i.e., 974 participants from wave 1 and 1,048 participants added in wave 2).

New to the second wave of data collection and repeated in the third wave of data collection, participants were asked information about any positive events that they had experienced that day in five domains: interpersonal interactions, at work, at home, network (something positive happened to someone else that the participant perceived as positive), and other. Participants were also asked to rate their levels of positive and negative affect. The current study used data from participants who completed both the MIDUS survey and the NSDE subproject and made use of data from both wave 2 (~2005) and wave 3 (~2015).

### Measures

#### Sociodemographic Covariates (Sex, Race, and Education)

Sex (0=male, 1=female) was included as a covariate because depression symptomatology and prevalence have been shown to vary based on sex, specifically lifetime prevalence of major depressive disorder is higher in females (APA, 2013). For example, women have been found to report more severe symptoms, more somatic and cognitive-affective symptoms, as well as higher rates of comorbid anxiety (Eid et al., 2019). Participants self-identified as African American and Black, Asian or Pacific Islander, Native American, White, and other. Race was included as a covariate because cultural differences may lead to differential depression symptom expression, such as increased somatic symptoms in certain cultures, and differential lifetime prevalence, with minority groups reporting higher prevalence rates (APA, 2013). Cell sizes for individual non-white racial categories were low, necessitating a dichotomized race variable coded as 0=white and 1=not white. It is important to note that the current study recognizes that the lives of historically marginalized individuals cannot be equated, and that this analytic decision does not reflect the diversity of experience across or within cultural groups. Education was coded as 0 (high school or less) or 1 (some college or more) and included as a covariate because education has been shown to be linked to mental health, with low education being associated with higher prevalence of mental illness (Araya et al., 2003).

#### Structural Covariates (Medication, Chronic Health Conditions, Anxiety Disorder)

##### Medication.

Participants were asked a range of questions about medication usage during the NSDE. One question asked if participants had taken anti-depressants or anti-anxiety medication during the course of data collection at waves 2 and 3 (0 = no, 1 = yes). This was included as a covariate because usage of anti-depressant medication may impact emotional regulation processes, with one study finding that it amplified observed MB effects (Bylsma et al., 2011).

##### Chronic health conditions.

Participants were asked a series of questions asking: “in the past 12 months, have you experienced or been treated for any of the following… [diabetes/hypertension/asthma/etc.]” where no = 0 and yes = 1. These were compiled into a composite count variable indicating the number of chronic conditions that each participant experienced at baseline. This was included as a covariate because previous research has found that older adults with four or more chronic conditions experience higher levels of stressor reactivity than older adults with fewer chronic conditions (Piazza et al., 2007), which may be related to positive event responsivity. Consistent with previous research by Piazza and colleagues, we removed depression and anxiety from the composite count variable and retained the following list of chronic conditions: cancer, heart trouble, lupus, heart attack, stroke, high blood pressure, stomach problems, constipation, ulcer, asthma, tuberculosis, other lung problems, joint and bone problems, sciatica, migraine, gum problems, teeth problems, diabetes, foot problems, hay fever, gall bladder problems, neurological conditions, skin problems, thyroid problems, and urinary problems. A count variable operationalized the number of chronic conditions across five groups consistent with previous research (0 = no chronic conditions, 1 = 1 chronic condition, 2 = 2 chronic conditions, 3 = 3 chronic conditions, 4 = 4 or more chronic conditions; Piazza et al., 2007).

##### Anxiety disorder.

Participants were asked 3 initial questions regarding how much they worry. If participants reported that 1) they worry more than most people, 2) worried every day, almost every day, or most days, and 3) reported that they worry about more than one thing at once, they then completed a 10-item scale asking them how often they experience a range of anxiety symptoms on a scale from 1 (most days) to 4 (never). If they did not meet these 3 pre-conditions, they did not complete the 10-item scale. Items included questions such as “in the past 12 months how often were you… restless because of your worry” and “tired easily because of your worry”. Anxiety (0=no anxiety, 1=anxiety) was included as a covariate because MB has been found to be specific to depression (Khazanov et al., 2019) and comorbid anxiety may obscure the unique impact of depression on affective responsivity.

#### Age

A continuous age variable centered at sample average baseline age (age at wave 2) was used as a covariate and in moderation analyses.

#### Depression

In the MIDUS survey, participants were asked if there was a period of 2 or more weeks in the past 12 months where they felt depressed. If they answered yes to this question, they completed a 7-item scale asking about symptoms of depression experienced during that two-week period based on DSM-III-R criteria (Wang et al., 2000). Differences between the DSM-III-R criteria and DSM-5 criteria for major depression are minimal, so this measure is comparable to modern standards of assessment (APA, 2013). Because the DSM-III-R stipulates a period of two or more weeks of either depressed mood or loss of interest as criterion A, if participants answered no to the previous question they were then asked if there was a period of two or more weeks in the past 12 months where they lost interest in most things. If they answered yes to this question, they completed a 6-item scale identical to the previous one except for one item asking if they “lost interest in most things”. Participants were also asked how much of each day they felt depressed/lost interest and how many days during the two weeks they felt that way. These items were used to construct a binary variable (0=no depression, 1=depression) where participants were coded yes = 1 (they experienced a depressive episode in the past 12 months) if they met the following criteria: 1) they answered yes to 4 or more of the binary questions (e.g., “did you [lose interest in most things/lose your appetite/etc.]?”), and 2) they reported feeling sad/depressed/lost interest that lasted “all day long” or “most of the day” and this occurred “everyday” or “almost every day”.

#### Daily Positive Events

At the end of each day of the NSDE, participants were asked if they experienced a positive event in any of five domains (interaction, work, home, network, other). We coded a dichotomous exposure variable where 0 = no positive event reported and 1 = at least one positive event reported each day.

#### Daily Affect

During the telephone interview each night, participants were asked to rate their levels of positive and negative affect using items based on the Positive and Negative Affect Schedule (PANAS; Watson et al., 1988). The scales consisted of 13 items for positive affect (e.g., “did you feel [enthusiastic/confident/attentive/etc.] today?”) and 14 items for negative affect (e.g., “did you feel [lonely/upset/irritable/etc.] today?”). Participants rated these items on a 5-point Likert-type scale ranging from 1 = none of the time to 5 = all of the time. For each day, an average across all 13 PA items was made to create a daily PA score and an average across all 14 NA items was made to create a daily NA score. This facilitated up to 8 average scores of PA and NA for each participant at both waves of assessment. Higher values indicated greater PA and greater NA. Following Cranford and colleagues (2006), NA and PA demonstrated adequate within- and between-person reliability (NA: within-person = 0.77, between-person=0.97; PA: within-person=0.86, between-person=0.99; Scott et al., 2020).

#### Affective Responsivity

Affective responsivity for each participant was operationalized as the slope of the positive event-affect associations across days using a multilevel modeling approach. Daily positive and negative affect scores were each regressed on the dichotomous positive event exposure variable. The intercept value represented the level of affect on days without a positive event and the slope value represented the change in affect from a non-positive event day to a day with a positive event. Higher intercept values reflected higher values of affect on non-positive event days. Higher slope values reflected greater affective responsivity to positive events.

### Analytic Plan

#### Descriptive Statistics

**Figure 1:**
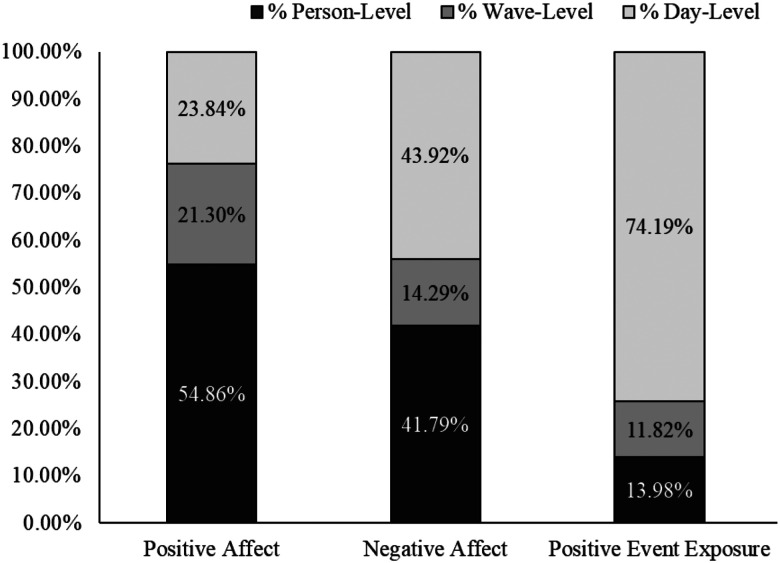
Variance Decompositions for Primary Study Variables *Note*. Values depicted reflect the proportion of variation across persons, waves, and days of assessment.

Means, frequencies, standard deviations, ranges, and bivariate correlations for all study variables, grouped into participants with and without depression were conducted first to evaluate distributions and assess relationships among primary study variables. Descriptive statistics are reported for both wave 2 and wave 3. Next, we conducted variance decompositions for all time-varying study variables (i.e., positive events, PA, NA) to parse out how much of the variation was accounted for at the day-, wave-, and person-levels in Figure 1. To do this, we used an empty (unconditional) multilevel model (MLM) to decompose variation at each level of analysis for people with depression and people without depression. Variance decompositions for PA, NA, and positive events were broken down into three-levels (i.e., day, wave, and person).

#### Statistics Addressing Primary Research Questions

To address our research questions, we used three-level MLMs (PROC MIXED; SAS Institute, 2013) to accommodate the nested structure of the data (8 days of daily diary data nested within two waves of assessment in ~2005 and ~2015 nested within 2,022 people). Maximum likelihood estimation was used due to missing data and attrition across days and waves of assessment. The MLMs for Hypotheses 1 and 2 are described below with the following equation and interpretation:

**Table:** 

*Level 1 (day)*:	${\text{Affect}}_{ijk}=\pi_{0ji}+\delta_{1ji}\left.{(\text{Day}}_{ijk}\right)+\delta_{2ji}({\text{PositiveEvent}}_{\text{tijk}})+i_{lk}$
*Level 2 (wave):*	$\pi_{0ji}=\beta_{00\text{i}}+\beta_{01\text{i}}(\left.{\text{Wave}}_{ji}\right)+\beta_{02\text{i}}\left.({\text{Medication}}_{ji}\right)+{\text{r}}_{0ji}$
	$\delta_{2ji}=\beta_{20\text{i}}+\beta_{21\text{i}}\left({\text{Wave}}_{ji}\right)$
*Level 3 (person):*	$\beta_{00i}=\gamma_{000}+\gamma_{001}\left.{(\text{Depression}}_{i}\right)+\gamma_{002}(\left.{\text{BaselineAge}}_{i}\right)+\gamma_{003}\left.{(Sex}_{i}\right)+\gamma_{004}\left({\text{Race}}_{i}\right)+\gamma_{005}\left({\text{Education}}_{i}\right)+\gamma_{006}\left({\text{Anxiety}}_{i}\right)+\gamma_{007}\left({\text{ChronicConditions}}_{i}\right)+u_{00i}$
	$\beta_{20\text{i}}=\gamma_{200}+\gamma_{201}\left({\text{Depression}}_{i}\right)+\gamma_{202}\left({\text{BaselineAge}}_{i}\right)+\gamma_{203}\left({\text{Depression}}_{i}*{\text{BaselineAge}}_{i}\right)$
	$\beta_{21\text{i}}=\gamma_{210}+\gamma_{211}\left({\text{Depression}}_{i}\right)$

Affect for person *i* at wave *j* on day *k* was regressed on positive event exposure (*δ*2_*ji*_) to model an estimate of affective responsivity. A linear trend across days of assessment (*δ*1_*ji*_) was included as a within-person (Level 1) covariate. Psychotropic medication use (β_02i_) was included as a within-person (Level 2) covariate. The presence of MB was tested with a positive event by depression two-way interaction term (γ_201_) examining whether the affective responsivity to positive events is amplified among people with depression compared to people without depression. To further adjust for sample heterogeneity and baseline factors, models were adjusted for wave 2 values of sex (γ_003_), race (γ_004_), education (γ_005_), anxiety (γ_006_), and chronic health conditions (γ_007_). These covariates were included in MLMs in a hierarchical fashion, with Model 1 only including primary main effects, interactions, and day- and wave-level trends. Model 2 added sociodemographic covariates (sex, race, education) and Model 3 added structural covariates (anxiety, medication, chronic conditions) to determine whether primary interactions of interest were robust to the inclusion of sociodemographic and structural factors, respectively.

To examine age differences in MB, we included a depression by positive event by age three-way interaction (i.e., γ_203_ in equation above). We ran two sets of MLMs for this research question, one with PA as the outcome and one with NA as the outcome. Follow-up analyses were run for significant omnibus interactions using ESTIMATE commands in SAS 9.4 to calculate simple slopes at −1 SD baseline age, the sample mean, and +1 SD baseline age, grouped by depression status. These are reported for significant two-way PositiveEvent*Depression interactions to capture MB, and significant three-way PositiveEvent*Depression*BaselineAge interactions to capture baseline age moderation.

To examine longitudinal changes in MB, we included a depression by positive event by wave three-way interaction term (i.e., γ_211_ in equation above). Similar to research question two, we ran two separate sets of MLMs, one with PA as the outcome and one with NA as the outcome. For this hypothesis, follow-up simple slopes analyses were run at wave 1 (2005) and at wave 2 (2015) grouped by depression status to unpack significant omnibus interaction effects. These are reported for significant two-way PositiveEvent*Depression interactions to capture MB, and significant three-way PositiveEvent*Depression*Wave interactions to capture longitudinal change.

Exploratory analyses were conducted by testing longitudinal changes in MB separately for comparatively younger adults in the sample, adults in midlife, and older adults for any instances of significant omnibus interactions. First, we computed age tertiles to break down the analytic sample into three age groups, with group 1 ranging from 35–51-years-old, group 2 ranging from 52–64-years-old, and group 3 ranging from 65–86 years old. We then re-ran analyses testing Hypothesis 2 individually for these three age groups in the analytic sample.

## Results

Descriptive statistics for the full sample grouped by wave and depression status are reported in Tables 1 and 2. Bivariate correlations between study variables are grouped into waves and reported in Tables 3 and 4. Additionally, we ran pairwise comparisons across depression status within waves. At baseline (wave 2), there were a higher percentage of female participants in the depression group (72.65%) than the not-depressed group (55.40%). Participants in the depression group (*M*age = 54.34, *SD* = 10.92) were also significantly younger than those who were not (*M*age = 59.12, *SD* = 12.16, *p* < .001), had lower PA (*M* = 2.20, *SD* = 0.89) than the rest of the sample (*M* = 2.78, *SD* = 0.66), and higher NA (*M* = 0.46, *SD* = 0.46) than the rest of the sample (*M* = 0.18, *SD* = 0.23). Both groups of participants had similar percentages of non-white participants (15.53%) and college-educated participants (68.83%). There were significantly more participants in the depression group who reported taking anti-depressants (30.97%) on any of the days of assessment during wave 2 than in the not-depressed group (12.78%) and who screened positive for anxiety (15.35%) than those in the not-depressed group (0.61%). Finally, the proportion of positive event days was significantly lower in the depressed group (66.74% of days) than the not-depressed group (71.22% of days). This pattern of results and statistical significance for differences across depression status remained identical at wave 3 for all variables except for the proportion of positive event days where participants no longer differed significantly by depression status and the full sample experienced positive events on approximately 82% of study days.

Unconditional MLMs showed significant between-person and within-person variation in PA, NA, and positive event exposure across days and waves of assessment (Figure 1). For PA, 54.86% of the variation was attributed to between-person individual differences, 21.30% was attributed to variation across waves, and 23.84% was attributed to variation across study days, unspecified sources of variation, and error. For NA, 41.79% of the variation was attributed to between-person individual differences, 14.29% to variation across waves, and 43.92% to variation across study days, unspecified sources of variation, and error. For positive event exposure, 13.98% of the variation was attributed to between-person individual differences, 11.82% to variation across waves, and 74.19% to variation across study days, unspecified sources of variation, and error.

### Baseline Age Differences in MB

**Figure 2:**
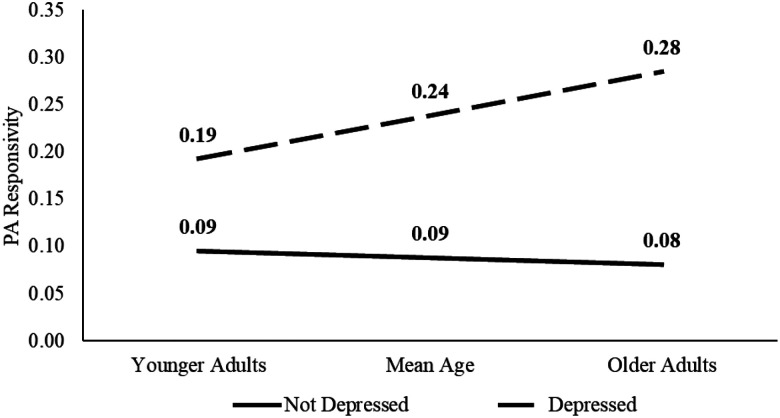
Cross-Sectional Baseline Age Differences in Positive Affective Responsivity to Positive Events *Note*. Values are from Table 5 Model 1’s significant positive event*depression*baseline age three-way interaction (Est. = 0.01, *SE* = 0.002, *p* = .03, 95% CI [0.00, 0.01]). Responsivity slopes plotted for not depressed and depressed groups at mean age and one standard deviation below and above the mean. This significant interaction was robust to sociodemographic variable and structural variable adjustment.

**Figure 3:**
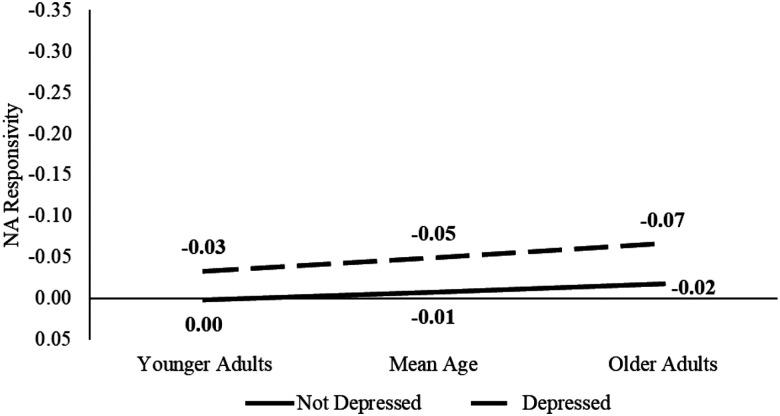
Cross-Sectional Baseline Age Differences in Negative Affective Responsivity to Positive Events *Note*. Values are from Table 6 Model 1’s non-significant positive event*depression*baseline age three-way interaction (Est. = −0.001, *SE* = 0.001, *p* = .60, 95% CI [0.00, 0.00]). Responsivity slopes plotted for not depressed and depressed groups at mean age and one standard deviation below and above the mean.

MLM parameter estimates for positive events predicting PA and NA outcomes represent the magnitudes of affective responsivity to positive events, defined statistically as the association between exposure to positive events and affect for the corresponding affective valence of interest. Figures 2 and 3 show graphs of the Model 1 results for PA and NA responsivity, respectively, across depression status at −1 SD baseline age, the sample mean, and +1 SD baseline age.

#### PA Models

When MLMs were run with PA entered as the outcome, the two-way interaction between positive event exposure and depression was significant (Est. = 0.15, *SE* = 0.02, *p* < .001, 95% CI [0.10, 0.20]; Model 1). The simple slope analyses estimating responsivity slopes across depression status characterized the presence of MB across all ages in the sample such that responsivity was higher among people who screened positive for depression (Est. = 0.2, *SE* = 0.02, *p* < .001; Model 1) than those who did not (Est. = 0.09, *SE* = 0.01, *p* < .001; Model 1).

The hypothesis for baseline age moderation was supported when predicting PA. Specifically, a significant three-way interaction between depression, positive event, and baseline age (Est. = 0.01, *SE* = 0.002, *p* = .03, 95% CI [0.00, 0.01]; Model 1) indicated the presence of cross-sectional baseline age differences in the magnitude of MB for PA. This effect was robust to the inclusion of sociodemographic covariates in Model 2 (Est. = 0.004, *SE* = 0.002, *p* = .04, 95% CI [0.00, 0.01]), and structural covariates in Model 3 (Est. = 0.01, *SE* = 0.002, *p* = .04, 95% CI [0.00, 0.01]). MB was strongest among older adults in the sample when compared to younger adults. Specifically, the simple slopes analyses revealed that, among younger adults (-1SD age, ~46-year-olds) in the sample, PA responsivity was higher for participants who screened positive for depression (Est. = 0.19, *SE* = 0.02, *p* < .001), compared to those who did not (Est. = 0.09, *SE* = 0.01, *p* < .001). This gap widened (approximately doubled in magnitude) for the comparatively older adults (+1SD age, ~70-year-olds), where participants who screened positive for depression again had higher PA responsivity (Est. = 0.28, *SE* = 0.04, *p* < .001) than those who did not (Est. = 0.08, *SE* = 0.01, *p* < .001). Figure 2 shows these findings from Model 1 visually, with participants who screened positive for depression displaying higher overall levels of PA responsivity to positive events than those who did not (i.e., MB) as well as the increasing gap in PA responsivity between the two groups (indicating cross-sectional baseline age differences). PA responsivity was more similar across baseline age for the non-depressed group, with older and younger adults reporting similar levels. In the depression group, however, older adults tended to report higher levels of PA responsivity than younger adults.

#### NA Models

When including NA as the model outcome, the results showed a different pattern from PA. Once again, the PositiveEvent*Depression interaction term was significant (Est. = −0.04, *SE* = 0.01, *p* < .01, 95% CI [-0.07, −0.02]; Model 1), indicating the presence of MB across ages. Simple slope analysis estimating responsivity slopes across depression status characterized the presence of MB across all ages in the sample such that responsivity was higher among people who screened positive for depression (Est. = −0.05, SE = 0.01, p < .001) than those who did not (Est. = −0.01, SE = 0.004, p = .07).

In contrast to the PA models, baseline age did not moderate the observed MB effects for NA (Model 1: Est. = −0.001, *SE* = 0.001, *p* = .60, 95% CI [0.00, 0.00]; Model 2: Est. = −0.001, *SE* = 0.001, *p* =. 48, 95% CI [0.00, 0.00]; Model 3: Est. = −0.0017, *SE* = 0.0012, *p* = .163, 95% CI [0.00, 0.00]).

### Longitudinal Changes in MB

#### PA Models

When considering PA as the outcome, the two-way interaction between positive event exposure and depression that operationalized MB was significant (Est. = 0.15, *SE* = 0.03, *p* < .001, 95% CI [0.10, 0.21]; Model 1), indicating the presence of MB.

The hypothesis for longitudinal change in MB was supported when predicting PA. Specifically, a PositiveEvent*Depression*Wave interaction term indicated significant longitudinal change in MB across the ten-year follow-up (Est. = −0.10, *SE* = 0.05, *p* = .05, 95% CI [−0.19, −0.01]; Model 1). When adjusting for sociodemographic variables in Model 2, this pattern of results and statistical significance held (Est. = −0.10, *SE* = 0.05, *p* = .04, 95% CI [−0.19, 0.00]). However, this moderation effect attenuated to marginal significance when structural covariates were included in Model 3 (Est. = −0.09, *SE* = 0.05, *p* = .08, 95% CI [−0.19, 0.01]). Simple slopes analysis revealed discrepant longitudinal trajectories depending on depression status. Participants who screened positive for depression tended to start higher in PA responsivity to positive events in 2005 (Est. = 0.22, *SE* = 0.03, *p* < .001) and decrease in responsivity over ten years (Est. = 0.19, *SE* = 0.04, *p* < .001). Participants in the not-depressed group, in contrast, showed an opposite trajectory. These participants started lower in PA responsivity in 2005 (Est. = 0.07, *SE* = 0.01, *p* < .001) and increased in responsivity over time (Est. = 0.14, *SE* = 0.01, *p* < .001).

#### NA Models

There were different patterns of results found when NA was entered as the outcome. Similar to PA, the two-way interaction between positive event exposure and depression that operationalized MB was significant (Est. = −0.04, *SE* = 0.01, *p* = .01, 95% CI [−0.07, −0.01]), indicating the presence of MB.

However, the PositiveEvent*Depression*Wave three-way interaction was not significant in Model 1 (Est. = 0.03, *SE* = 0.03, *p* = .25, 95% CI [−0.02, 0.08]). The magnitude of the three-way interaction term estimate increased in Model 2 (Est. = −0.03, *SE* = 0.03, *p* = .22, 95% CI [−0.02, 0.08]) and Model 3 (Est. = 0.05, *SE* = 0.04, *p* = .07, 95% CI [0.00, 0.10]) but never reached statistical significance, indicating that there were no evidence of significant changes in MB for NA responsivity across the ten-year follow-up.

### Exploratory Analysis: Exploring Age Differences in Longitudinal Change in MB

We also conducted exploratory analysis to extend research question 2 and evaluate whether the significant longitudinal changes in MB in the PA models varied as a function of age group. Age tertiles stratified the analytic sample into younger adults (N = 646, Mean age = 44.89 years, SD = 4.32, Range = 35–51), adults in midlife (N = 717, Mean age = 58.06 years, SD = 3.74, Range = 52–64), and older adults (N = 658, Mean age = 72.69 years, SD = 5.84, Range = 65–86). The following results report findings for PA models stratified by each of these age groups.

#### Younger Adults

Among younger adults, the PositiveEvent*Depression*Wave three-way interaction was not significant in Model 1 (Est. = −0.09, *SE* = 0.07, *p* = .22, 95% CI [−0.22, 0.05]), after adjustment for sociodemographics in Model 2 (Est. = −0.09, *SE* = 0.07, *p* = .21, 95% CI [−0.22, 0.05]), or after adjustment for structural covariates in Model 3 (Est. = −0.09, *SE* = 0.07, *p* = .22, 95% CI [−0.24, 0.06]). This indicates that there were no significant differences in longitudinal change in PA responsivity for the comparatively younger adults in the sample across depression status.

#### Adults in Midlife

Among adults in midlife, the PositiveEvent*Depression*Wave three-way interaction was also not significant in Model 1 (Est. = −0.05, *SE* = 0.08, *p* = .52, 95% CI [−0.21, 0.10]), after adjustment for sociodemographics in Model 2 (Est. = −0.05, *SE* = 0.08, *p* = .50, 95% CI [−0.21, 0.10]), or after adjustment for structural covariates in Model 3 (Est. = −0.03, *SE* = 0.09, *p* = .76, 95% CI [−0.20, 0.14]). This indicates that there were no significant differences in longitudinal change in PA responsivity for the adults in midlife across depression status.

#### Older Adults

Among older adults, the PositiveEvent*Depression*Wave interaction term was significant in Model 1 (Est. = −0.38, *SE* = 0.12, *p* < .01, 95% CI [−0.62, −0.13]). When adjusting for sociodemographic covariates in Model 2 (Est. = −0.37, *SE* = 0.12, *p* < .01, 95% CI [−0.61, −0.13]) and structural covariates in Model 3 (Est. = −0.48, *SE* = 0.14, *p* < .001, 95% CI [−0.76, −0.19]) the pattern of results and statistical significance remained consistent, indicating the presence of depression status differences in longitudinal change trajectories of PA responsivity for older adults in the sample. This estimate was robust to adjustment for both sociodemographic and structural variables. Simple slopes analysis unpacked the discrepant longitudinal trajectories across depression status. Participants who screened positive for depression tended to start higher in PA responsivity to positive events in 2005 (Est. = 0.34, *SE* = 0.06, *p* < .001) and decrease in responsivity over ten years resulting in a non-significant slope in 2015 (Est. = 0.05, *SE* = 0.10, *p* = .61). Participants in the not-depressed group, in contrast, showed an opposite trajectory. These participants started lower in PA responsivity in 2005 (Est. = 0.06, *SE* = 0.02, *p* < .001) and increased in responsivity over time (Est. = 0.14, *SE* = 0.03, *p* < .001).

These exploratory results suggest the significant longitudinal change in MB observed in the PA models may be specific to comparatively older adults in the present study’s analytic sample. No significant change in MB across the 10-year follow-up was observed for the younger adult and midlife age groups.

## Discussion

The present study provides the first examination of age moderation and longitudinal changes in MB in a national adult lifespan sample. Because age has important implications for ER (Charles & Piazza, 2024), it is crucial to develop understanding of how these daily affective dynamics in depression are operating at different points in the lifespan. The present study revealed complex age patterns in affective responsivity for people with and without depression that provide novel insight into unique vulnerabilities of older adulthood. MB was robustly present for both PA and NA, replicating previous MB findings using the NSDE (Mukherjee et al., 2023). Below we discuss the cross-sectional age differences and longitudinal changes in MB that extends research in affective science from a lifespan developmental perspective.

### Cross-Sectional Baseline Age Differences in MB

Older adults with depression exhibited the strongest pattern of MB, with PA responsivity slopes larger than younger age groups and indicative of possible emotion dysregulation. In line with SAVI (Charles, 2010; Charles & Piazza, 2024), these results suggest that depression may act as a chronically sustained stressor that counteracts strengths in ER associated with older adulthood. These findings were specific to PA, with the magnitude of MB being more similar across ages for NA. Interestingly, most previous research only detected MB for NA (e.g., Bylsma et al., 2008), and not PA. This discrepancy with the current results could be related to differences in the relative severity of NA items across studies. The NSDE measure includes items on psychological distress in addition to items from the PANAS. Given the NSDE consists of a predominantly healthy sample, the inclusion of these items may limit variation in NA composite scores.

Socioemotional Selectivity Theory (SST) states that older adults’ goals and motivations prioritize meaning-making and close social connection because their future time perspective is shorter than younger adults (Carstensen et al., 1999). However, the presence of a shorter future time perspective may mean something different for an individual with depression, especially in the presence of symptoms of hopelessness or suicidality. The disengagement and demotivation that characterizes depression may interfere with meaning-making and goal-directed behaviors, which could be particularly distressing for older adults, leading to the increased PA responsivity in the results. Additionally, older adults may be especially vulnerable to the social isolation and inhibition that often accompanies depression.

Previous research on “fragile positive affect” has also identified that highly variable PA (i.e., increased responsivity) combined with low-baseline PA, which is characteristic of depression, may be a risk factor for a variety of negative health outcomes (Ong & Ram, 2017). This previous research would suggest that older adults with depression may be especially vulnerable to a range of harmful outcomes beyond the disorder itself. Thus, it may be especially valuable to target PA responsivity in older adults in clinical settings. Highly variable PA, or more fragile PA, is also more subject to external influences (Ong & Ram, 2017). Older adults may disproportionately benefit from interventions whose goal is to instill a more internal source of positive emotions that is less subject to external influences, such as those common in the field of positive psychology, as one avenue to target PA responsivity.

### Longitudinal Changes in MB

Results showed that people with depression tended to start high in PA responsivity and decrease over time, while people without depression tended to start low in PA responsivity and increase over time. While increased responsivity to positive events may reflect the outcome of maladaptive ER processes, that explanation may not fully explain the reasons behind these findings. Previous lifespan developmental research has identified the presence of a “positivity bias” associated with age, where older adults tend to exhibit a preference for positive information such that they attend to and remember positive information more than younger adults (Carstensen & DeLiema, 2018). Because attending to a given stimulus is an important intermediary step in the ER process described earlier, increased attention to positive stimuli may lead to increased responsivity to positive stimuli. People with depression, however, tended to decrease in responsivity to positive events over time, which may be attenuating capacity for a positivity bias. One explanation is that depression directly counteracts the trajectory towards more attention to positive stimuli.

After adjusting for anxiety, chronic health conditions, and medication use, the longitudinal change trajectories of PA responsivity were no longer significantly different across depression status. We conducted follow-up sensitivity analyses with each structural covariate added into Model 3 independently and found that the three-way interaction term was robust to adjustment for anxiety disorder and chronic conditions, but attenuated to marginal significance when controlling for concurrent antidepressant or antianxiety medication usage. Thus, medication use may be partially explaining some of the variation in longitudinal change of PA responsivity. However, additional research is needed to investigate mechanisms for why this would be the case. Baseline age also informed the trajectory of changes in MB across the 10-year follow-up. In exploratory analyses, only the comparatively older adults (i.e., 65–86 years) exhibited significant longitudinal changes in MB that were robust to adjustment for medication. Personalized intervention efforts (e.g., Hamburg & Collins, 2010) interested in modifying PA responsivity may benefit from these results to better pinpoint at which ages responsivity may be especially valuable to target (e.g., older adults).

### Limitations and Future Directions

The strengths of this study must be interpreted alongside its limitations. The sample consisted predominantly of healthy, college-educated, non-Hispanic white adults in the US, which is not fully representative of the US population. Future work should recruit samples that are more representative of the sociodemographic composition of US adults. Future research may also benefit from additional measurement occasions per day of assessment in ecological momentary assessment designs (e.g., Cerino & Hooker, 2019) that can isolate and characterize relationships between positive events and affective reactions within and across study days to more precisely assess MB in daily life.

## Conclusion

Older adults are uniquely vulnerable to emotion dysregulation associated with depression. The present study’s findings help build a better understanding of how depression manifests in daily life and informs more tailored approaches to promote mental health across developmental phases of the adult lifespan (Hamburg & Collins, 2010).

## Figures and Tables

**Figure 4:**
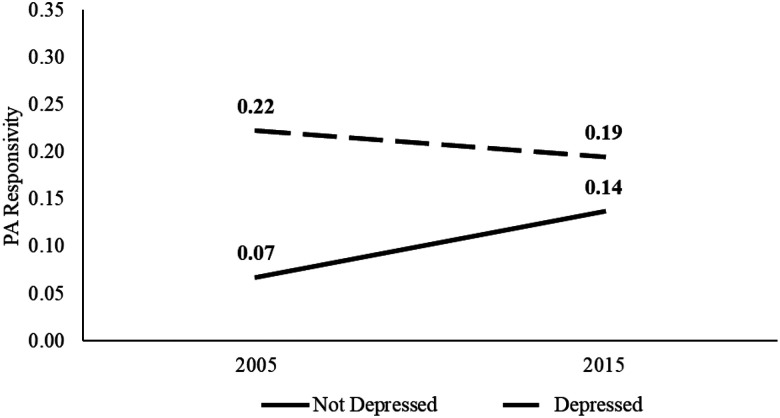
Longitudinal Changes in Positive Affective Responsivity to Positive Events *Note*. Values are from Table 7 Model 1’s significant positive event*depression*wave three-way interaction (Est. = −0.10, *SE* = 0.05, *p* = .04, 95% CI [−0.19, −0.01]). Responsivity slopes plotted for not depressed and depressed groups at wave 2 (~2005) and ten years later at wave 3 (~2015). This significant interaction was robust to sociodemographic variable adjustment, but attenuated to marginal significance when adjusting for structural variables.

**Figure 5:**
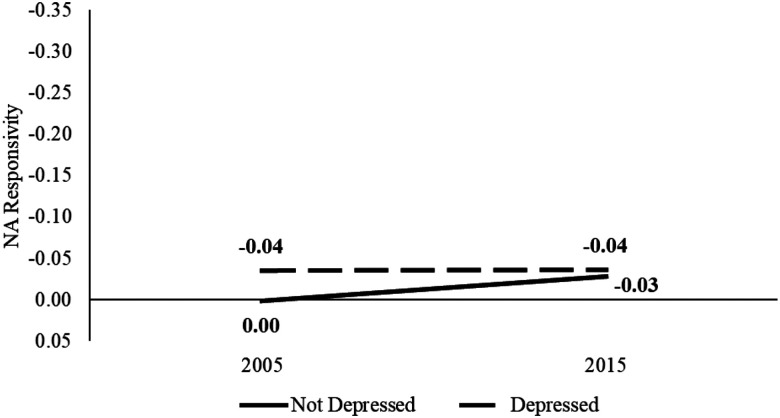
Longitudinal Changes in Negative Affective Responsivity to Positive Events *Note*. Values are from Table 8 Model 1’s non-significant positive event*depression*wave three-way interaction (Est. = 0.03, *SE* = 0.03, *p* = .25, 95% CI [−0.02, 0.08]). Responsivity slopes plotted for not depressed and depressed groups at wave 2 (~2005) and ten years later at wave 3 (~2015).

**Table 1:** Sample Descriptives at Wave 2

Variable	Range	Full Sample (2,022)	Not Depressed (1,807)	Depressed (215)	*t* or *χ*^*2*^	*p*
Age	35, 86	58.61 (12.12)	59.12 (12.16)	54.34 (10.92)	5.51	<.001
Female (%)	0, 1	57.22	55.4	72.56	23.12	<.001
Non-White (%)	0, 1	15.53	15.55	15.35	0.01	.940
College (%)	0, 1	68.83	69.35	64.49	2.11	.147
0 Chronic Conditions (%)	0, 1	19.83	22.95	10.23	38.89	<.001
1 Chronic Condition (%)	0, 1	21.96	20.26	21.4	—	—
2 Chronic Conditions (%)	0, 1	18.1	19.69	14.88	—	—
3 Chronic Conditions (%)	0, 1	15.28	13.74	12.56	—	—
4+ Chronic Conditions (%)	0, 1	24.83	23.35	40.93	—	—
Medication (%)	0, 1	14.58	12.78	30.97	37.11	<.001
Anxiety (%)	0, 1	2.18	0.61	15.35	196.11	<.001
Depression (%)	0, 1	10.63	—	—	—	—
Positive Affect	0.04, 4	2.72 (0.71)	2.78 (0.66)	2.20 (0.89)	11.67	<.001
Negative Affect	0, 2.54	0.21 (0.28)	0.18 (0.23)	0.46 (0.46)	−14.59	<.001
Positive Events (% of study days)	0, 8	70.75	71.22	66.74	2.27	.024

*Note*. Sample sizes for each group are reported in parentheses next to each description in column headings. Means are reported for all continuous variables with standard deviations reported next to each mean in parentheses. Frequencies for categorical variables are expressed as percentages of the sample where indicated. P-values in each column after the descriptives for each wave represent the significance of tests of group comparisons between participants in the sample who are not-depressed vs. depressed for each variable indicated. The p-values reported for 0 Chronic Conditions row represent the significance of the omnibus chi-square tests of depression status differences in membership across all five categories of the Chronic Conditions variable.

**Table 2:** Sample Descriptives at Wave 3

Variable	Range	Full Sample (1,236)	Not Depressed (1,100)	Depressed (136)	*t* or *χ*^*2*^	*p*
Age	47, 95	67.67 (10.34)	67.98 (10.34)	65.14 (9.66)	3.01	.003
Female (%)	0, 1	57.20	55.36	72.06	13.78	<.001
Non-White (%)	0, 1	15.84	16.16	13.24	0.78	.378
College (%)	0, 1	74.47	75.05	69.85	1.72	.190
0 Chronic Conditions (%)	0, 1	21.76	22.91	12.5	28.44	<.001
1 Chronic Condition (%)	0, 1	24.11	24.09	24.26	—	—
2 Chronic Conditions (%)	0, 1	19.58	19.82	17.65	—	—
3 Chronic Conditions (%)	0, 1	15.7	16.27	11.03	—	—
4+ Chronic Conditions (%)	0, 1	18.85	16.91	34.56	—	—
Medication (%)	0, 1	18.1	15.56	37.84	32.63	<.001
Anxiety (%)	0, 1	1.94	0.36	14.71	130.76	<.001
Depression (%)	0, 1	11.00	—	—	—	—
Positive Affect	0.21, 4	2.68 (0.69)	2.73 (0.66)	2.27 (0.84)	7.13	<.001
Negative Affect	0, 2.86	0.18 (0.24)	0.16 (0.21)	0.33 (0.42)	−7.33	<.001
Positive Events (% of study days)	0, 8	81.97	82.33	78.97	1.60	.109

*Note*. Sample sizes for each group are reported in parentheses next to each description in column headings. Means are reported for all continuous variables with standard deviations reported next to each mean in parentheses. Frequencies for categorical variables are expressed as percentages of the sample where indicated. P-values in each column after the descriptives for each wave represent the significance of tests of group comparisons between participants in the sample who are not-depressed vs. depressed for each variable indicated. The p-values reported for 0 Chronic Conditions row represent the significance of the omnibus chi-square tests of depression status differences in membership across all five categories of the Chronic Conditions variable.

**Table 3:** Bivariate Correlations at Wave 2 (~2005)

Variable	*n*	1	2	3	4	5	6	7	8	9	10	11
1. Age	2,021	—										
2. Female	2,021	−0.02	—									
3. Race	2,016	−0.05*	0.05*	—								
4. College	2,017	−0.11***	−0.07**	0.097***	—							
5. Chronic Conditions	1,954	0.21***	0.14***	0.071**	−0.08***	—						
6. Medication	1,564	−0.01	0.12***	−0.06*	0.00	0.24***	—					
7. Positive Event	2,021	0.07**	0.05*	0.097***	0.22***	−0.03	0.08**	—				
8. Positive Affect	2,021	0.20***	−0.01	−0.015	−0.04	0.18***	−0.16***	0.10***	—			
9. Negative Affect	2,021	−0.16***	0.07**	0.10***	−0.03	0.18***	0.19***	−0.01	−0.50***	—		
10. Anxiety	2,021	−0.07**	0.05*	0.06**	−0.07**	0.16***	0.12***	−0.05*	−0.16***	0.24***	—	
11. Depression	2,021	−0.12***	0.11***	−0.00	−0.03	0.21***	0.15***	−0.05*	−0.25***	0.31***	0.31***	—

Note.

* *p* < .05.

** *p* < .01.

*** *p* <.001.

**Table 4:** Bivariate Correlations at Wave 2 (~2005)

Variable	*n*	1	2	3	4	5	6	7	8	9	10	11
1. Age	1,220	—										
2. Female	1,220	−0.03	—									
3. Race	1,216	−0.06*	0.06*	—								
4. College	1,218	−0.06*	−0.10***	−0.10***	—							
5. Chronic Conditions	1,197	0.20***	0.19***	0.07*	−0.07*	—						
6. Medication	1,044	−0.07*	0.10**	−0.06*	0.01	0.24***	—					
7. Positive Event	1,171	0.12***	0.08**	−0.17***	0.22***	0.01	−0.03***	—				
8. Positive Affect	1,174	0.15***	−0.03	0.02	−0.05	−0.15***	−0.19***	0.158***	—			
9. Negative Affect	1,174	−0.12***	0.04	0.12***	−0.00	0.20***	0.20***	−0.07*	−0.54***	—		
10. Anxiety	1,220	−0.07*	0.06*	0.02	−0.07*	0.14***	0.09**	−0.05	−0.16***	0.17***	—	
11. Depression	1,220	−0.09**	0.11***	−0.03	−0.04	0.21***	0.18***	−0.05	−0.20***	0.21***	0.33***	—

Note.

* *p* < .05.

** *p* < .01.

*** *p* <.001.

**Table 5:** Cross-Sectional Baseline Age Differences in Positive Affective Responsivity

Variables	Model 1: Unadjusted		Model 2: Sociodemographic Variable Adjustment		Model 3: Structural Variable Adjustment	
	Estimate (*SE*)	95% CI	Estimate (*SE*)	95% CI	Estimate (*SE*)	95% CI
Intercept	2.7045 (.0174)***	[2.6704, 2.7386]	2.7299 (.0338)***	[2.6636, 2.7963]	2.8853 (.0417)***	[2.8035, 2.9671]
Day	.0032 (.0011)**	[0.0010, 0.0055]	.0032 (.0011)**	[0.0010, 0.0055]	.0022 (.0012)	[−0.0002, 0.0047]
Wave	−.0544 (.0166)**	[−0.0870, −0.0218]	−.0554 (.0167)**	[−0.0880, −0.0227]	−.0578 (.0179)**	[−0.0929, −0.0227]
Race	—	—	.0043 (.0400)	[−0.0742, 0.0828]	.0283 (.0435)	[−0.0570, 0.1135]
College	—	—	−.0594 (.0316)	[−0.1214, 0.0026]	−.0830 (.0339)**	[−0.1496, −0.0164]
Female	—	—	.0248 (.0292)	[−0.0325, 0.0821]	.0537 (.0309)	[−0.0070, 0.1144]
Chronic conditions	—	—	—	—	−.0641 (.0114)***	[−0.0864, −0.0417]
Medication	—	—	—	—	−.4718 (.1126)***	[−0.6927, −0.2509]
Anxiety	—	—	—	—	−.1519 (.0351)***	[−0.2207, −0.0831]
Positive event (PE)	.0877 (.0078)***	[0.0725, 0.1030]	.0885 (.0078)***	[0.0732, 0.1038]	.0907 (.0086)***	[0.0740, 0.1075]
Depression	−.6293 (.0529)***	[−0.7330, −0.5256]	−.6187 (.0533)***	[−0.7232, −0.5142]	−.4398 (.0605)***	[−0.5585, −0.3211]
PE*Depression	.1510 (.0247)***	[0.1025, 0.1994]	.1466 (.0249)***	[0.0978, 0.1954]	.1529 (.0271)***	[0.0998, 0.2060]
Baseline Age (BA)	.0098 (.0013)***	[0.0072, 0.0125]	.0095 (.0014)***	[0.0069, 0.0122]	.0116 (.0015)***	[0.0085, 0.0146]
PE*BA	−.0006 (.0006)	[−0.0019, 0.0007]	−.0006 (.0006)	[−0.0019, 0.0007]	−.0005 (.0007)	[−0.0019, 0.0009]
Depression*BA	−.0065 (.0045)	[−0.0153, 0.0024]	−.0050 (.0045)	[−0.0139, 0.0039]	−.0061 (.0049)	[−0.0156, 0.0034]
PE*Depression*BA	.0046 (.0021)*	[0.0005, 0.0087]	.0043 (.0021)*	[0.0002, 0.0084]	.0046 (.0022)*	[0.0002, 0.0090]

*Note*. Model 1: N=2,021, Observations=22,442. Model 2: N=2,012, Observations=22,348. Model 3: N=1,711, Observations=18,794.

* *p* < .05.

** *p* < .01.

*** *p* <.001.

**Table 6:** Cross-Sectional Baseline Age Differences in Negative Affective Responsivity

Variables	Model 1: Unadjusted		Model 2: Sociodemographic Variable Adjustment		Model 3: Structural Variable Adjustment	
	Estimate (*SE*)	95% CI	Estimate (*SE*)	95% CI	Estimate (*SE*)	95% CI
Intercept	.2341 (.0070)***	[0.2204, 0.2478]	.2211 (.0124)***	[0.1968, 0.2453]	.1640 (.0139)***	[0.1366, 0.1913]
Day	−.0148 (.0006)***	[−0.0160, −0.0135]	−.0147 (.0006)***	[−0.0160, −0.0135]	−.0143 (.0007)***	[−0.0156, −0.0130]
Wave	−.0146 (.0062)*	[−0.0268, −0.0024]	−.0138 (.0062)*	[−0.0260, −0.0016]	−.0090 (.0064)	[−0.0216, 0.0036]
Race	—	—	.0663 (.0143)***	[0.0383, 0.0942]	.0579 (.0141)***	[0.0301, 0.0856]
College	—	—	−.0090 (.0113)	[−0.0311, 0.0131]	−.0015 (.0111)	[−0.0232, 0.0201]
Female	—	—	.0143 (.0104)	[−0.0061, 0.0347]	.0010 (.0101)	[−0.0187, 0.0207]
Chronic conditions	—	—	—	—	.0228 (.0037)***	[0.0155, 0.0301]
Medication	—	—	—	—	.0621 (.0119)***	[0.0387, 0.0855]
Anxiety	—	—	—	—	.2316 (.0367)***	[0.1596, 0.3036]
Positive event (PE)	−.0078 (.0043)	[−0.0161, 0.0006]	−.0075 (.0043)	[−0.0159, 0.0008]	−.0100 (.0046)*	[−0.0190, −0.0009]
Depression	.2772 (.0205)***	[0.2371, 0.3173]	.2778 (.0204)***	[0.2379, 0.3177]	.1800 (.0214)***	[0.1380, 0.2220]
PE*Depression	−.0419 (.0135)**	[−0.0684, −0.0154]	−.0449 (.0136)***	[−0.0716, −0.0183]	−.0402 (.0147)**	[−0.0689, −0.0115]
Baseline Age (BA)	−.0020 (.0005)***	[−0.0030, −0.0010]	−.0019 (.0005)***	[−0.0029, −0.0009]	−.0023 (.0005)***	[−0.0034, −0.0012]
PE*BA	−.0008 (.0004)*	[−0.0015, −0.0001]	−.0009 (.0004)*	[−0.0015, −0.0002]	−.0010 (.0004)**	[−0.0018, −0.0003]
Depression*BA	.0018 (.0017)	[−0.0016, 0.0052]	.0021 (.0017)	[−0.0013, 0.0054]	.0031 (.0017)	[−0.0003, 0.0064]
PE*Depression*BA	−.0006 (.0011)	[−0.0028, 0.0016]	−.0008 (.0011)	[−0.0031, 0.0014]	−.0017 (.0012)	[−0.0041, 0.0007]

*Note*. Model 1: N=2,021, Observations=22,441. Model 2: N=2,012, Observations=22,347. Model 3: N=1,711, Observations=18,793.

* *p* < .05.

** *p* < .01.

*** *p* <.001.

**Table 7:** Longitudinal Changes in Positive Affective Responsivity

Variables	Model 1: Unadjusted		Model 2: Sociodemographic Variable Adjustment		Model 3: Structural Variable Adjustment	
	Estimate (*SE*)	95% CI	Estimate (*SE*)	95% CI	Estimate (*SE*)	95% CI
Intercept	2.7294 (.0176)***	[2.6949, 2.7640]	2.7863 (.0332)***	[2.7212, 2.8515]	2.8875 (.0411)***	[2.8069, 2.9681]
Day	.0031 (.0011)**	[0.0009, 0.0053]	.0031 (.0011)**	[0.0009, 0.0053]	.0022 (.0012)	[−0.0002, 0.0045]
Wave	−.1189 (.0214)***	[−0.1608, −0.0770]	−.1216 (.0215)***	[−0.1636, −0.0795]	−.1185 (.023)***	[−0.1636, −0.0735]
Race	—	—	.0010 (.0387)	[−0.0749, 0.0769]	.0160 (.0421)	[−0.0666, 0.0986]
College	—	—	−.0865 (.0307)**	[−0.1466, −0.0263]	−.1112 (.0331)***	[−0.1760, −0.0463]
Female	—	—	.0034 (.0285)	[−0.0524, 0.0593]	.0214 (.0303)	[−0.0380, 0.0807]
Chronic conditions	—	—	—	—	−.0340 (.0105)**	[−0.0546, −0.0134]
Medication	—	—	—	—	−.1641 (.0342)***	[−0.2312, −0.0971]
Anxiety	—	—	—	—	−.5108 (.1099)***	[−0.7263, −0.2953]
Positive event (PE)	.0677 (.0090)***	[0.0500, 0.0854]	.0687 (.0090)***	[0.0510, 0.0864]	.0717 (.0101)***	[0.0519, 0.0914]
Depression	−.6662 (.0516)***	[−0.7674, −0.5649]	−.6598 (.0519)***	[−0.7616, −0.5580]	−.4777 (.0603)***	[−0.5960, −0.3594]
PE*Depression	.1548 (.0267)***	[0.1025, 0.2072]	.1507 (.0268)***	[0.0982, 0.2033]	.1557 (.0302)***	[0.0965, 0.2149]
PE*Wave	.0691 (.0162)***	[0.0374, 0.1008]	.0711 (.0162)***	[0.0393, 0.1029]	.0656 (.0172)***	[0.0319, 0.0994]
Depression*Wave	.1562 (.0632)*	[0.0324, 0.2800]	.1550 (.0631)*	[0.0313, 0.2788]	.1100 (.0685)	[−0.0242, 0.2442]
PE*Depression*Wave	−.0967 (.0468)*	[−0.1884, −0.0050]	−.0955 (.0469)*	[−0.1873, −0.0036]	−.0890 (.0501)	[−0.1872, 0.0093]

*Note*. Model 1: N=2,191, Observations=23,691. Model 2: N=2,180, Observations=23,573. Model 3: N=1,853, Observations=19,885.

* *p* < .05.

** *p* < .01.

*** *p* <.001.

**Table 8:** Longitudinal Changes in Negative Affective Responsivity

Variables	Model 1: Unadjusted		Model 2: Sociodemographic Variable Adjustment		Model 3: Structural Variable Adjustment	
	Estimate (*SE*)	95% CI	Estimate (*SE*)	95% CI	Estimate (*SE*)	95% CI
Intercept	.2222 (.0071)***	[0.2083, 0.2360]	.2022 (.0120)***	[0.1786, 0.2258]	.1596 (.0137)***	[0.1328, 0.1864]
Day	−.0146 (.0006)***	[−0.0158, −0.0135]	−.0146 (.0006)***	[−0.0158, −0.0134]	−.0142 (.0007)***	[−0.0155, −0.0130]
Wave	.0155 (.0094)	[−0.0029, 0.0339]	.0160 (.0094)	[−0.0026, 0.0345]	.0193 (.0098)*	[0.0000, 0.0386]
Race	—	—	.0618 (.0136)***	[0.0352, 0.0884]	.0572 (.0135)***	[0.0308, 0.0837]
College	—	—	−.0004 (.0107)	[−0.0215, 0.0206]	.0055 (.0106)	[−0.0153, 0.0263]
Female	—	—	.0177 (.0099)	[−0.0018, 0.0372]	.0065 (.0097)	[−0.0125, 0.0255]
Chronic conditions	—	—	—	—	.0149 (.0034)***	[0.0083, 0.0215]
Medication	—	—	—	—	.2345 (.0353)***	[0.1654, 0.3036]
Anxiety	—	—	—	—	.0691 (.0115)***	[0.0466, 0.0916]
Positive event (PE)	.0011 (.0049)	[−0.0085, 0.0107]	.0011 (.0049)	[−0.0085, 0.0107]	.0000 (.0054)	[−0.0106, 0.0106]
Depression	.2906 (.0199)***	[0.2516, 0.3295]	.2897 (.0198)***	[0.2509, 0.3285]	.193 (.0216)***	[0.1507, 0.2353]
PE*Depression	−.0369 (.0145)*	[−0.0653, −0.0085]	−.0390 (.0145)**	[−0.0675, −0.0105]	−.037 (.0162)*	[−0.0688, −0.0052]
PE*Wave	−.0296 (.0087)***	[−0.0468, −0.0125]	−.0294 (.0088)***	[−0.0466, −0.0122]	−.0293 (.0092)**	[−0.0474, −0.0113]
Depression*Wave	−.0938 (.0273)***	[−0.1473, −0.0402]	−.0961 (.0273)***	[−0.1496, −0.0425]	−.086 (.0288)**	[−0.1424, −0.0295]
PE*Depression*Wave	.0289 (.0253)	[−0.0208, 0.0785]	.0309 (.0254)	[−0.0188, 0.0806]	.0484 (.0269)	[−0.0043, 0.1010]

*Note*. Model 1: N=2,191, Observations=23,688. Model 2: N=2,180, Observations=23,570. Model 3: N=1,853, Observations=19,882.

* *p* < .05.

** *p* < .01.

*** *p* <.001.
